# Early Detection and Classification of Gibberella Zeae Contamination in Maize Kernels Using SWIR Hyperspectral Imaging and Machine Learning

**DOI:** 10.3390/s26061834

**Published:** 2026-03-14

**Authors:** Kaili Liu, Shiling Li, Wenbo Shi, Zhen Guo, Xijun Shao, Yemin Guo, Jicheng Zhao, Xia Sun, Nortoji A. Khujamshukurov, Fangling Du

**Affiliations:** 1 College of Agricultural Engineering and Food Science, Shandong University of Technology, No. 266 Xincun Xilu, Zibo 255049, China; mykaili0905@gmail.com (K.L.); lion719500371@outlook.com (S.L.); suegyu123@163.com (W.S.); 15224267767@163.com (X.S.); sunxia2151@sina.com (X.S.); 2 Shandong Muyang New Energy Co., Ltd., Fulai Industrial Park, Rizhao 276800, China; 3 State Key Laboratory of Macromolecular Drugs and Large-Scale Preparation, Shandong Key Laboratory of Applied Technology for Protein and Peptide Drugs, School of Pharmaceutical Sciences and Food Engineering, Liaocheng University, Liaocheng 252000, China; guozhen@lcu.edu.cn; 4 Department of Biotechnology, Tashkent Institute of Chemical Technology, Tashkent 100011, Uzbekistan; nkhujamshukurov@mail.ru

**Keywords:** maize kernel, *Gibberella zeae*, hyperspectral imaging, spectral preprocessing, feature wavelength selection, classification model

## Abstract

Early-stage fungal contamination in maize kernels is difficult to identify visually and it can cause severe quality and safety risks during storage and transportation. Short-wave infrared (SWIR) hyperspectral imaging offers a rapid, non-destructive approach by capturing chemical information related to water, proteins, and lipids. This study investigates the early detection and classification of *Gibberella zeae* contamination in maize kernels using SWIR hyperspectral imaging combined with machine learning. Two maize varieties were artificially inoculated and cultured under controlled conditions, followed by hyperspectral data collection over six contamination stages. Various preprocessing techniques including standard normal variate (SNV), second derivative (SD), multiplicative scatter correction (MSC), and derivatives were evaluated to enhance data quality. Feature wavelength selection was performed using successive projections algorithm (SPA), competitive adaptive reweighted sampling (CARS), and uninformative variable elimination (UVE), significantly reducing redundancy and improving classification performance. Multiple models, including linear discriminant analysis (LDA), multilayer perceptron (MLP), support vector machine (SVM), a convolutional neural network (CNN), long short-term memory (LSTM) network, and a hybrid architecture Transformer that integrated a CNN, a LSTM network, and a Transformer (abbreviated as CLT), were constructed for both binary (healthy vs. contaminated) and multiclass classification tasks. Specifically, the multiclass task consisted of six contamination stages corresponding to contamination time from Day 0 to Day 5. The best binary classification task accuracy of 100% was achieved using SNV-preprocessed data with the MLP model. For multiclass classification task, the SD-preprocessed LDA model reached a test accuracy of 92.56%. Combined with appropriate preprocessing, feature selection and modeling, these results demonstrate that hyperspectral imaging is a powerful tool for the non-destructive, early-stage identification of fungal contamination in maize kernels, offering strong support for food safety and quality monitoring.

## 1. Introduction

Maize is one of the most important cereal, feed, and industrial crops worldwide, with widespread cultivation and a critical role in agricultural production. China ranks the second place globally, following the United States, in terms of both maize production and consumption. According to data from the Food and Agriculture Organization of the United Nations and the National Bureau of Statistics of China, the country’s maize production showed a fluctuating upward trend from 2013 to 2024, maintaining a stable share in global maize output. In 2024, China’s maize planting area reached 67.11 million hectares, and total production amounted to 294.92 million tons, surpassing those of wheat and rice, thus underscoring maize’s leading position among food crops in China [1].

Beyond its agricultural importance, maize serves as a raw material for over 3000 industrial products across food, pharmaceutical, and chemical sectors [2], demonstrating enormous potential for economic growth and significant implications for food security and social stability.

However, maize kernels are particularly susceptible to fungal contamination during storage and transportation due to their large germ proportion, strong hygroscopicity, and rich nutrient content [3]. Under high temperature and humidity conditions, fungi on the kernel surface can rapidly proliferate, leading to widespread mold contamination [4]. It is estimated that fungal spoilage causes a post-harvest loss of approximately 21 million tons of grain annually in China, while globally, mold contamination affects about 25% of total grain production [5]. Mold deterioration not only destroys the nutritional value and germination capacity of maize but also results in color loss, foul odors, and even complete loss of edible and commercial value [6]. Among the fungal species, Aspergillus flavus is particularly prevalent [7], producing aflatoxin B1 (AFB_1_), a highly carcinogenic and toxic compound with structural stability that renders it resistant to degradation during food processing [5,8,9,10,11]. In addition, *Gibberella zeae* is another major pathogen affecting maize kernels. This fungus degrades the external quality and nutritional content of maize and produces mycotoxins that pose health risks to humans and animals [12]. Contamination by *Gibberella zeae* during storage and transportation further accelerates mold deterioration, leading to significant economic losses [13,14]. To address these threats, China has issued national standards, including GB 1353-2018 [15] or maize inspection, which explicitly lists mold contamination as a critical evaluation parameter. Currently, there are two major methods for assessing mold in maize kernels. The first is manual inspection, where inspectors visually examine a 100 g maize sample and determine the proportion of moldy kernels by weight. According to the national standard GB 1353-2018, a maize sample is categorized as acceptable if the moldy kernel mass fraction does not exceed 2.0%. However, this method is time-consuming (30–40 min per sample), subjective, labor-intensive, and prone to errors, especially during large-scale inspections [16] The second method is microbial detection, which involves culturing fungi with reagents and specialized equipment. Although more accurate, this approach requires large sample volumes, and it is laborious and destructive, which it is unsuitable for high-throughput non-destructive testing [17,18,19]. Given the limitations of traditional mold detection methods and the inadequacy of machine vision techniques in recognizing early-stage mold, spectral technology has emerged as a promising alternative for agricultural quality assessment [20,21]. Spectroscopic techniques, including hyperspectral imaging [22], Raman spectroscopy [23], and molecular fluorescence spectroscopy [24], enable rapid and non-destructive evaluation of both the external and internal quality of agricultural products. Short-wave infrared (SWIR) hyperspectral imaging provides vibrational information in the 1000–2500 nm range, targeting specific functional groups such as O–H (water, carbohydrates), N–H (proteins), and C–H (lipids) [12,13,14]. SWIR imaging combined with multivariate statistical analysis enables detection of hydrogen-containing organic compounds like aflatoxins [25]. Meanwhile, visible and near-infrared (VNIR) hyperspectral imaging captures physical characteristics such as defects, color changes, and texture degradation due to fungal contamination [26]. Compared to conventional methods, hyperspectral imaging offers significant advantages such as higher sensitivity, non-destructive analysis, faster measurements, and the ability to simultaneously extract both two-dimensional spatial and three-dimensional spectral information. This dual capability supports both qualitative and quantitative assessments of food products.

Recent studies have demonstrated the feasibility of hyperspectral imaging combined with chemometrics or machine learning for detecting fungal contamination and mycotoxin risks in grains, including maize kernels. The efficient determination of toxigenic fungi and aflatoxin contamination is achieved by these spectroscopic techniques, and specific spectral features are successfully extracted to overcome background interference in dried fruits and nuts [27,28]. Furthermore, single-cereal-kernel analysis is facilitated by near-infrared HSI to address the heterogeneity of contamination batches, wherein highly contaminated kernels are discriminated with high accuracies exceeding 90% for fungal infection and 80% for mycotoxins [29]. Additionally, voluminous HSI data are integrated with sophisticated chemometric methods and machine learning algorithms, and specific spectral signatures of fungal contamination in diverse grains and oilseeds are accurately identified to enhance food safety and quality assurance [30]. SWIR hyperspectral imaging are particularly informative for capturing chemical changes associated with water absorption, protein alteration, and lipid degradation during fungal growth. Meanwhile, deep-learning-based spectral or spectral–spatial modeling has shown promising performance in complex classification tasks. However, early-stage contamination staging (time-resolved multiclassification) and systematic comparisons among preprocessing, wavelength selection, and multiple model families remain insufficiently explored for *Gibberella zeae* contamination in maize kernels. In recent years, hyperspectral imaging has achieved considerable success in detecting crop composition and identifying diseases and pests [31,32,33]. Accordingly, this study aims to apply hyperspectral imaging technology to the detection and classification of fungal contamination in maize kernels, providing scientific support for grain quality assurance and food safety. In SWIR hyperspectral modeling, spectral preprocessing is commonly adopted to suppress instrumental noise, baseline drift, and scattering effects, thereby improving signal consistency. In addition, variable (wavelength) selection is often employed to reduce redundancy and collinearity in high-dimensional spectra, which can enhance computational efficiency and model robustness. Model selection further determines how effectively the spectral information is transformed into discriminative patterns; therefore, comparing conventional machine learning models with deep learning architectures can provide complementary insights into both interpretability and representation capability.

In this study, maize kernels were inoculated with *Gibberella zeae* under controlled conditions. Hyperspectral imaging data were subsequently acquired across six distinct contamination stages (Day 0 to 5). We conducted both a binary classification task (distinguishing healthy from contaminated kernels) and a multiclass classification task (identifying the specific stages of contamination). Furthermore, the spectral variations induced by fungal proliferation were systematically analyzed, and the impacts of various spectral preprocessing techniques and model combinations on classification performance were comprehensively evaluated. In this work, we positioned our contribution as a systematic application study. Specifically, (i) we establish a controlled and reproducible SWIR hyperspectral imaging protocol for maize kernel contamination assessment, (ii) benchmark representative preprocessing and wavelength-selection strategies and quantify their influence on binary detection and time-staged multiclass recognition, and (iii) provide practical guidance on selecting data-processing pipelines and model families for early-stage fungal contamination monitoring.

## 2. Materials and Methods

### 2.1. Sample Preparation

In this study, two maize varieties, Zhengdan 958 and Ruiyan 809, were selected. All samples were collected from a supermarket in Zibo, China, ensuring that each maize kernel was uniform in size and maturity and free from insect damage or disease. To ensure experimental reproducibility, a total of 4000 maize kernels were selected, with 2000 kernels assigned to the control group and 2000 kernels to the contaminated group. The samples were first soaked in 75% ethanol for 1 min, rinsed three times with sterile water, and then dried under sterile conditions.

The fungal strain used for contamination was *Gibberella zeae*, obtained from the China National Gene Bank. The strain was cultured on PDA medium at 28 °C for 5 days. After incubation, the spores were collected and diluted to a concentration of 1 × 10^6^ CFU/mL. The diluted spore suspension was then evenly inoculated onto the maize kernels designated for the contaminated group, while the control group kernels were treated with sterile water. All samples were incubated at 27 °C and 90% relative humidity for 5 days to provide optimal conditions for fungal growth. This sample preparation process established a solid experimental foundation for subsequent hyperspectral imaging detection. This study also designed two classification tasks: a binary classification task and a six-class classification task. The binary classification task aimed to distinguish between healthy and *Gibberella zeae*-contaminated maize kernels, while the six-class classification task categorized contaminated kernels based on contamination time, ranging from day 0 to day 5. The selection of these six stages was designed to monitor the progressive physicochemical transformations during the dynamic early window of fungal contamination under controlled conditions (27 °C and 90% RH). Day 0 represents the healthy control group. From Day 1 to Day 5, the inoculated *Gibberella zeae* undergoes a distinct growth cycle, advancing from initial spore germination to rapid mycelial proliferation and extensive colonization. These biological processes lead to a systematic, day-by-day degradation of the maize kernel matrix, involving the consumption of proteins, lipids, and carbohydrates, along with continuous fluctuations in moisture content. To ensure these six stages are biologically and spectrally distinct, an initial spectral analysis was performed, which reveals a consistent and significant hierarchical shift in reflectance intensity and absorption depths across the six-day period. This confirms that each 24 h interval captures a unique and measurable state of fungal-induced biochemical alteration.

### 2.2. Hyperspectral Image Acquisition

**Figure 1:**
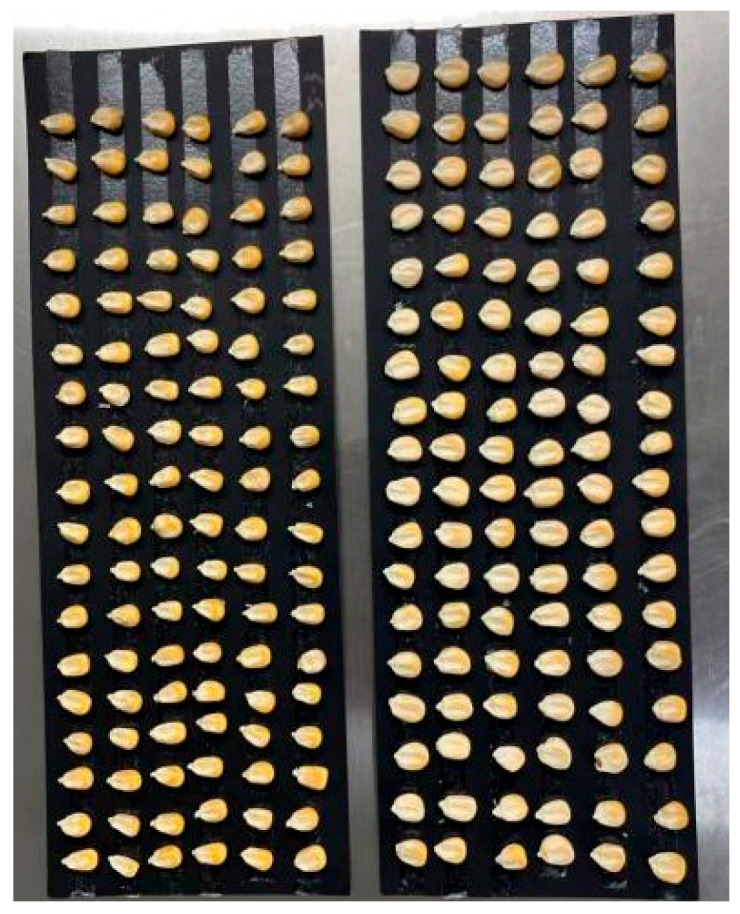
Sample placement.

A SWIR hyperspectral imaging system was used for maize kernel imaging, operating in the 900–2500 nm near-infrared range. The system includes a computer, imaging camera, precision lenses, illumination sources, spectrometer, stepper motor, and displacement platform. The internal components were housed in a dark chamber to minimize light interference. Detailed component information is listed in Table 1, and the system schematic is shown in Figure 1.

For sample preparation, the equipment used included a high-pressure steam sterilizer, mold incubator, clean bench, electronic balance, biological microscope, electric blast drying oven, pipettes, and an ultrapure water system, as summarized in Table 2. All instruments were selected to ensure experimental reliability. During hyperspectral imaging, exposure time was set to 5.5 ms, and the displacement stage speed to 11.5 mm/s, ensuring clear images with minimal blurring. Samples were continuously moved under the camera in line-scan mode. All imaging was conducted in a dark room to eliminate external light interference. System calibration was performed using standard white (99% reflectance) and dark reference images.

**Figure 2:**
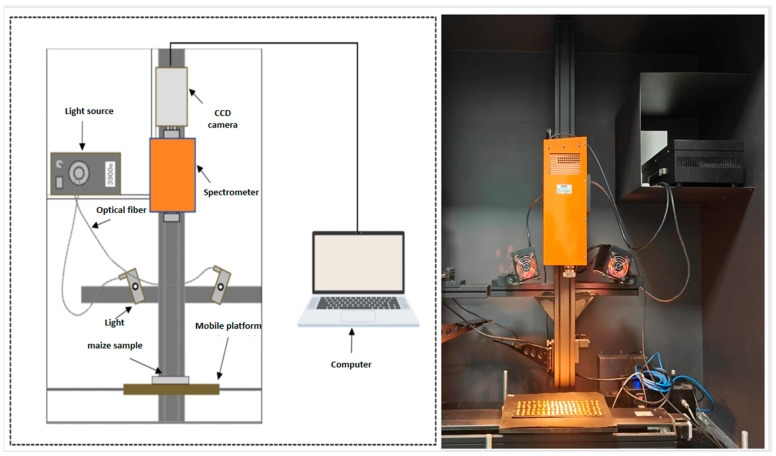
Physical diagram of hyperspectral imaging system.

A total of 40 hyperspectral images were collected, with each containing 100 maize kernels: 20 images from the contaminated group and 20 from the control group. To prevent sample movement during scanning, kernels were fixed on a black cardboard surface (Figure 2). After imaging, regions of interest (ROI) for individual kernels were extracted for spectral data analysis. Data acquisition and preprocessing were conducted using HSI Analyzer software (version 2015; ITT Visual Information Solutions, Boulder, CO, USA). Subsequent feature selection and model development were performed with Python 3.8.10, while Origin 2018 was used for data visualization. This workflow ensured efficient and accurate processing of hyperspectral data for subsequent analysis.

### 2.3. Spectral Preprocessing Methods

Raw hyperspectral data often contain random noise, baseline drift, and scattering effects, which can compromise the relationship between spectral features and sample characteristics. Therefore, various preprocessing techniques, including Savitzky–Golay (SG) smoothing [6], standard normal variate (SNV) [7], multiplicative scatter correction (MSC) [8], first derivative (FD) [9], and second derivative (SD) [10], were employed to correct the spectral data. Specifically, SG smoothing suppresses high-frequency noise while preserving the overall shape of absorption features. SNV standardizes each spectrum to reduce multiplicative scatter effects and improve sample-to-sample comparability, whereas MSC corrects baseline offsets and scatter-induced variations by referencing each spectrum to an estimated ideal spectrum. In addition, FD and SD emphasize local spectral changes and can enhance subtle absorption features potentially associated with contamination-related chemical variations [12]. These preprocessing approaches aimed to enhance signal quality by reducing noise, correcting baseline shifts, and minimizing scattering interference, thus ensuring high-quality input for subsequent modeling efforts. In this study, these preprocessing methods were used to mitigate instrument noise, baseline drift, and scattering effects commonly observed in SWIR hyperspectral data, thereby improving the robustness of subsequent wavelength selection and classification.

### 2.4. Feature Wavelength Selection Methods

To reduce data dimensionality and focus on the most informative spectral features, three feature selection methods were used: successive projections algorithm (SPA) [34], competitive adaptive reweighted sampling (CARS) [35], and uninformative variable elimination (UVE) [36]. Specifically, SPA selects a small subset of wavelengths with minimal collinearity, improving model stability and reducing redundancy [37,38]. CARS iteratively samples and retains variables with strong predictive contribution by monitoring model error during the selection process. UVE removes uninformative variables by evaluating the stability/importance of each wavelength and discarding those with low reliability. These algorithms aimed to identify wavelengths that captured the most significant variance related to fungal contamination, thereby improving model performance and computational efficiency.

These selection strategies reduce the dimensionality of hyperspectral data and enable a consistent comparison of how compact versus broader wavelength subsets affect binary detection and time-staged multiclass recognition.

### 2.5. Model Development

LDA constructs a classification model by maximizing the separation between different classes while minimizing within-class variance in a reduced-dimensional space. LDA assumes that data follow a normal distribution and that the covariance matrices of different classes are equal, but it remains robust against noise and redundant information. The MLP is a feedforward neural network that employs multiple fully connected layers and nonlinear activation functions. It learns complex mappings from input features to output classes through backpropagation and gradient descent, making it suitable for hyperspectral data classification and feature extraction tasks. The combined PCA–LDA method integrates unsupervised and supervised dimensionality reduction techniques. PCA first reduces data dimensionality by capturing the principal variance components, while LDA subsequently enhances class separation in the lower-dimensional space, improving classification stability and performance. SVM is a machine learning technique based on statistical learning theory. It seeks an optimal hyperplane that maximizes the margin between classes. For nonlinear data, SVM uses kernel functions to map inputs into high-dimensional spaces, facilitating linear separability while balancing model complexity and generalization. This hybrid model integrates convolutional neural networks, long short-term memory networks, and Transformer architectures. Convolutional neural networks extract local spatial features, long short-term memory networks model temporal dynamics and sequence dependencies, and Transformers capture global contextual information through self-attention mechanisms. The combination of these network architectures enables powerful spatiotemporal feature modeling capabilities, making it particularly effective for processing complex, high-dimensional hyperspectral data. LDA seeks linear projections that maximize between-class separation relative to within-class variance, while SVM constructs a maximum-margin decision boundary and can model nonlinearity through kernel functions. MLP learns nonlinear mappings via stacked fully connected layers optimized by backpropagation. For deep learning models, CNN extracts local patterns from spectral or spectral–spatial inputs through convolutional filters, LSTM captures sequential dependencies and long-range relationships in ordered spectral inputs, and Transformers model global dependencies using self-attention mechanisms. Finally, CLT is a hybrid architecture integrating CNN-based feature extraction, LSTM-based sequence modeling, and Transformer-based global attention to enhance representation learning.

**Figure 3:**
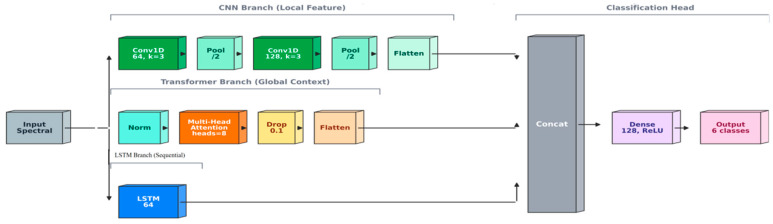
Architecture of the CNN–LSTM–Transformer (CLT) model for hyperspectral classification.

To improve clarity and reproducibility, the network architectures of CNN, LSTM, Transformer, and the hybrid CLT model are illustrated in Figure 3, including the input format, major blocks, and output layers.

### 2.6. Model Training and Hyperparameter Settings

The proposed CLT model consists of three parallel branches, including CNN, LSTM, and Transformer modules. A schematic illustration is shown in Figure 3, and the detailed architecture configuration is summarized in Table 3.

## 3. Results and Discussion

### 3.1. Spectral Analysis of Healthy and Moldy Maize Kernels Under Different Preprocessing Methods

**Figure 4:**
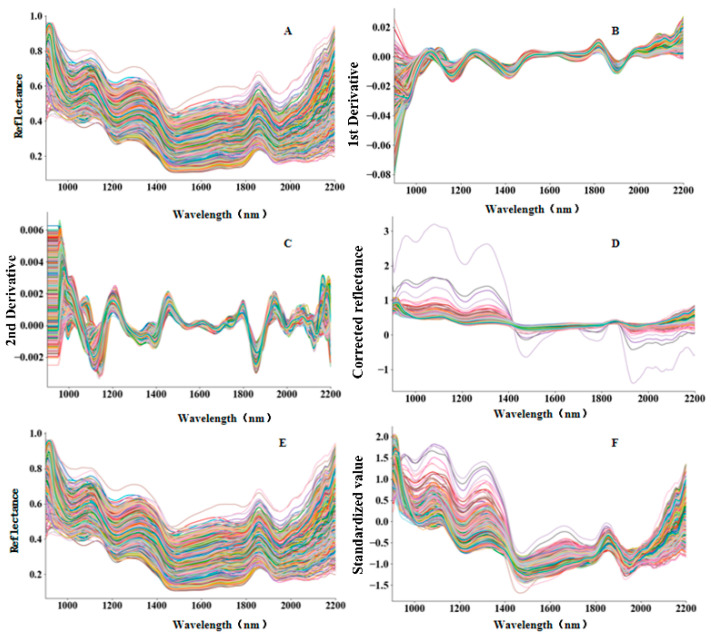
Spectral profiles of healthy and *Gibberella zeae*-contaminated maize kernels (900–2200 nm) under different preprocessing methods: (**A**) raw reflectance spectra, (**B**) FD spectra, (**C**) SD spectra, (**D**) MSC-corrected spectra, (**E**) SG smoothed reflectance spectra (**F**) SNV–transformed spectra.

Figure 4 presents the spectral profiles of maize kernels in the SWIR range (900–2200 nm) under different preprocessing methods. The raw spectra (Figure 4A) are expressed as reflectance and show typical absorption characteristics in the SWIR region, while also exhibiting baseline variability caused by illumination and scattering differences among kernels. After first-derivative (FD) preprocessing (Figure 4B), the spectra are transformed into derivative values, which naturally include both positive and negative amplitudes as FD emphasizes local slope changes and suppresses baseline effects. The second-derivative (SD) spectra (Figure 4C) further enhance subtle local absorption features and reduce baseline drift, resulting in smaller-amplitude oscillations around zero, which are characteristic of derivative spectra.

For scatter correction, multiplicative scatter correction (MSC) (Figure 4D) adjusts each spectrum relative to a reference spectrum, reducing multiplicative and additive scattering effects; therefore, the corrected spectra are no longer strictly constrained to the original reflectance range. Savitzky–Golay (SG) smoothing (Figure 4E) reduces high-frequency noise while preserving the overall reflectance scale and spectral shape. Standard normal variate (SNV) preprocessing (Figure 4F) standardizes each spectrum by centering and scaling, producing dimensionless standardized values that commonly span both positive and negative ranges. Overall, these preprocessing methods reduce noise and scattering-related variability to different extents and provide more robust spectral representations for subsequent feature selection and classification.

**Figure 5:**
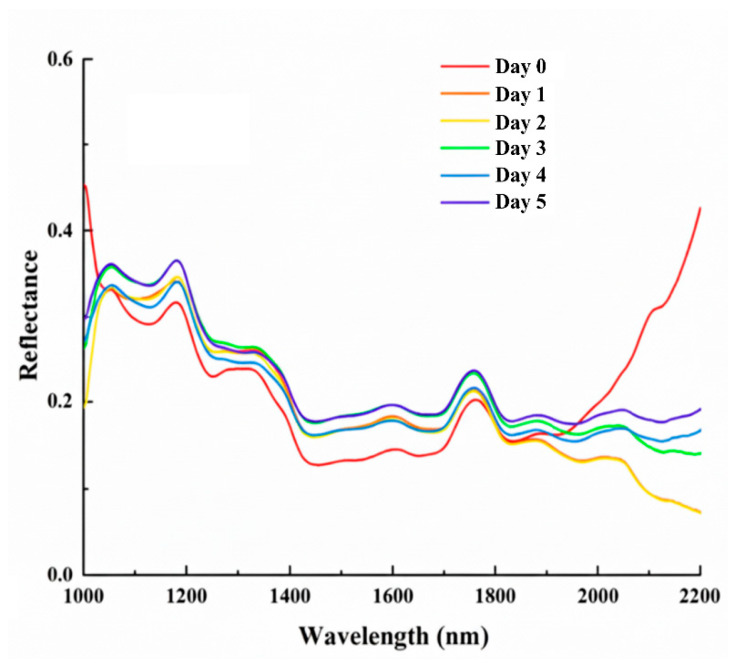
Average reflectance spectra of maize kernels at different contamination stages (D0–D5).

While Figure 4 illustrates the overall spectral distribution and the impact of various preprocessing techniques, the specific spectral divergence between different contamination stages is further clarified in Figure 5. As observed in the average spectra, the Day 0 (Healthy) kernels exhibit a distinctive profile compared to the infected samples, particularly in the late SWIR region. For the contaminated kernels (Day 1–Day 5), the progressive shifts in reflectance intensity are chemically linked to the degradation of starch and proteins, as well as changes in moisture content (O–H bonds near 1450 nm and 1940 nm) induced by *Gibberella zeae* proliferation. These nuanced spectral signatures at each stage provide the physical basis for the high accuracy achieved by the subsequent classification models.

### 3.2. Development and Evaluation of Binary and Multiclass Classification Models Based on Different Preprocessing Methods

In this study, several full-spectrum classification models were developed to identify moldy maize kernels by combining both raw spectral data and data preprocessed using various techniques, including SG, SNV, FD, SD, and MSC. The classification algorithms applied were LDA, Principal component analysis–linear discriminant analysis (PCA-LDA), SVM, CLT, and MLP. Based on the results shown in Table 4, the performance of each preprocessing method was compared across binary and six-class classification tasks to determine the most suitable preprocessing strategy.

In the six-class classification task, the SD-preprocessed dataset achieved the best performance, with the LDA model yielding a test accuracy of 92.56%, and the MLP model achieving 90.70%. The high accuracy of the SD method suggests that it effectively enhances class separability for multiclass mold contamination stages. For the two-class classification task all preprocessing methods led to significant improvements in model performance. However, SNV stood out as the most effective, with all models—except PCA-LDA—achieving 100% accuracy on both training and test sets (PCA-LDA achieved 99.88% test accuracy). This indicates that SNV preprocessing substantially improved data stability and model discriminative power for distinguishing between healthy and moldy kernels. Overall, SD was identified as the optimal preprocessing method for multiclass classification, while SNV performed best for binary classification. These methods were therefore selected for use in subsequent modeling and feature extraction processes.

**Figure 6:**
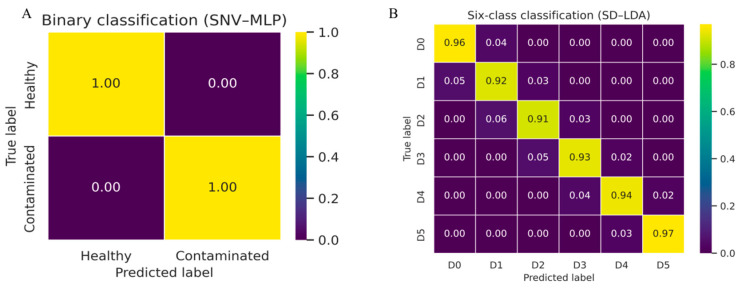
Normalized confusion matrices of the optimal models. (**A**) Binary classification using SNV-preprocessed data with the MLP model, showing perfect separation between healthy and contaminated maize kernels. (**B**) Six-class classification using SD-preprocessed data with the LDA model, where most misclassifications occur between adjacent contamination stages (D0–D5).

To further illustrate the classification behavior of the optimal models, Figure 6 presents the normalized confusion matrices for both the binary and six-class classification tasks. The binary model (SNV–MLP) achieved perfect separation between healthy and contaminated kernels, while the six-class model (SD–LDA) shows that most misclassifications occur between adjacent contamination stages.

### 3.3. Development of Binary and Multiclass Classification Models Based on Feature Wavelengths

Due to the three-dimensional nature of hyperspectral imaging data, the dataset contains a large volume of information across numerous spectral bands, making direct processing computationally intensive and time-consuming. To address this challenge, dimensionality reduction is often applied to eliminate redundant data and improve processing efficiency. In this study, three feature wavelength selection methods—SPA, CARS, and UVE—were employed to extract key wavelengths for both binary and six-class classification tasks involving maize kernels. After selecting the feature wavelengths, samples from the calibration and test sets were processed using these three methods to obtain the final set of representative spectral variables. These were subsequently used to develop classification models using five algorithms: Linear discriminant analysis (LDA), PCA-LDA, SVM, MLP, a and a hybrid model integrating CNN, LSTM, and Transformer architectures.

#### 3.3.1. Feature Wavelength Extraction

**Figure 7:**
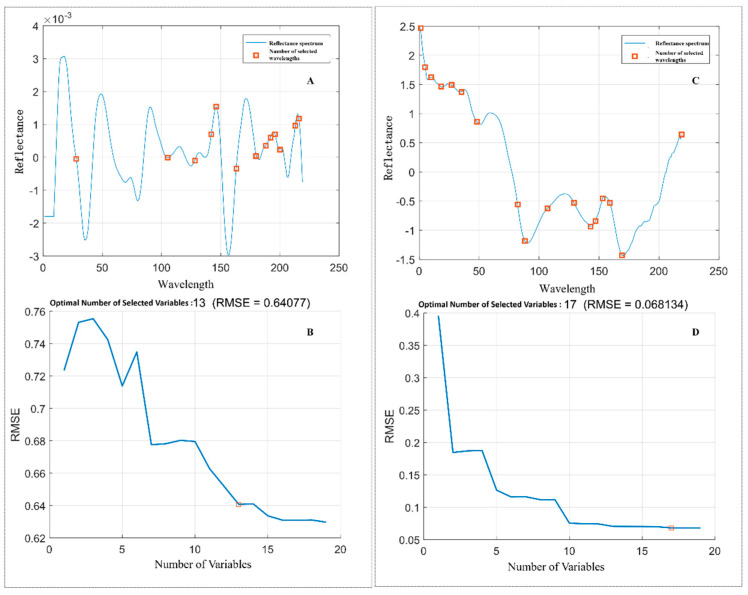
Root means square error of SPA and feature wavelength selection (**A**) RMSE of SPA for six-class task (**B**) feature wavelength selection of SPA for six-class task (**C**) RMSE of SPA for two-class task (**D**) feature wavelength selection of SPA for two-class task.

Figure 7 illustrates the root mean square error (RMSE) trends and the feature wavelength selection process using the SPA for both binary and six-class classification tasks. As the number of selected wavelengths increases, the RMSE consistently decreases and eventually stabilizes, indicating a reduction in variability and an improvement in model performance. In the six-class task (Figure 7A,B), the spectral reflectance initially shows notable fluctuations due to sample variability and potential noise or irrelevant variables, while RMSE is high when only a few wavelengths are selected. As more wavelengths are added, RMSE steadily declines, and when 13 wavelengths are selected, the error curve levels off, suggesting that further additions do not substantially improve accuracy. A similar trend is observed in the binary classification task (Figure 7C,D), where reflectance variability diminishes with more selected wavelengths, and RMSE rapidly decreases before stabilizing at around 17 wavelengths. Overall, the SPA-based selection process effectively enhances model accuracy and stability by identifying the most informative spectral bands, thereby validating its utility for both classification scenarios. The detailed wavelength-selection processes of CARS and UVE are provided in the Supplementary Materials Figures S1 and S2, while the main text focuses on the selected feature sets and downstream classification performance.

Figure S1 illustrates the CARS-based wavelength selection process and the associated RMSE evolution. For the six-class task (Figure S1A–C), RMSE decreases rapidly during the early iterations and becomes stable once approximately 40 wavelengths are retained, indicating that redundant variables have been removed and the selected subset reaches a plateau in predictive performance. For the two-class task (Figure S1D–F), a similar trend is observed, with RMSE stabilizing after approximately 30 wavelengths and reduced signal fluctuation. Overall, the RMSE stabilization provides a practical criterion for determining an adequate wavelength subset under CARS.

Figure S2 shows the UVE-based evaluation of variable importance for the six-class (A) and two-class (B) tasks. The yellow curve represents the t-values of all wavelengths, whereas the red curve indicates the wavelengths retained after UVE filtering. Large fluctuations in the initial t-values suggest the presence of uninformative or unstable variables, while the retained set becomes more stable after filtering. UVE retained 186 and 183 wavelengths for the six-class and two-class tasks, respectively, and the final subsets were further reduced to 151 and 140 wavelengths, demonstrating effective redundancy removal for subsequent classification.

The detailed wavelength-selection processes of CARS and UVE are provided in the Supplementary Materials Figures S1 and S2, and the complete lists of selected wavelengths for all methods are provided in Table S1. The main text focuses on the selected feature sets and downstream classification performance.

#### 3.3.2. Development and Comparison of Classification Models Based on Feature Wavelengths

Results of maize kernel two-class and six-class task models under different feature wavelength selection methods Table 5 presents the classification results of maize kernels for both binary and six-class tasks using different feature wavelength selection methods. The results lead to the following observations: For the six-class classification task, the LDA model using wavelengths selected by the UVE method achieved the best performance, with a training accuracy of 95.38% and a testing accuracy of 91.63%. This indicates that UVE effectively identifies informative wavelengths and significantly improves classification accuracy. In comparison, models based on SPA and CARS showed similar performance in the LDA and PCA-LDA frameworks, with test accuracies generally ranging from 73% to 79%, suggesting limited ability to distinguish multiple categories. The SVM model performed poorly in the six-class task across all methods, indicating its limitations in handling complex multiclass scenarios. CLT model achieved high training accuracy (95.17%) with UVE-selected features but experienced a drop to 87.79% in test accuracy, indicating a tendency toward overfitting.

In the two-class classification task, all models achieved high accuracy across different wavelength selection methods. However, the CLT model under the CARS method performed poorly, while the best overall performance was achieved by models using UVE-selected wavelengths.

In summary, the UVE method outperformed others in the six-class classification task, especially in combination with LDA and MLP models. In the two-class classification task differences among methods were relatively minor, but models based on UVE-selected wavelengths still achieved the highest overall accuracy. The selected feature wavelengths are expected to relate to chemical variations induced by fungal growth, such as changes in water-related O–H absorptions and compositional alterations associated with proteins (N–H) and lipids/carbohydrates (C–H). Different selection methods may yield varied results because they impose different sparsity and stability constraints: SPA tends to select a small, low-collinearity subset that may be insufficient for fine-grained staging; CARS is sensitive to sampling and model-error criteria and may vary across tasks; UVE retains a broader set of informative variables, which can preserve stage-related information and improve robustness in multiclass classification.

In summary, the UVE method outperformed others in the six-class classification task, especially in combination with LDA and MLP models. The specific wavelengths selected by UVE, along with those selected by SPA and CARS, are listed in Table S1 in the Supplementary Materials.

### 3.4. In-Depth Discussion and Comparison with Related Work

The feature wavelengths selected by UVE, SPA, and CARS (Table S1) can be associated with specific biochemical changes induced by *Gibberella zeae* contamination. Water-related O–H absorptions around 1450 nm (e.g., 1446, 1440 nm) and 1930–1940 nm (e.g., 1930, 1936 nm) were consistently selected, attributed to increased moisture during fungal growth [38]. Protein-related N–H absorptions in the 1500–1600 nm (e.g., 1551, 1557 nm) and 2050–2150 nm regions (e.g., 2058, 2080 nm) were also selected, linked to fungal protein synthesis [38,39]. Lipid and carbohydrate-related C–H absorptions in the 1700–1800 nm region (e.g., 1756, 1762, 1774, 1780 nm) were abundantly represented, associated with degradation of starch and lipids during fungal metabolism [40]. Two-dimensional correlation spectroscopy analysis reveals that fungal contamination induces stepwise consumption of compounds: soluble sugars → carbohydrates → starch → complex organics [39], explaining why multiple wavelength regions are required for fine-grained staging. The consistent selection of these key wavelengths across methods demonstrates that SWIR hyperspectral imaging can detect biochemical changes before visible symptoms appear [38,39,40].

### 3.5. Discussion

The results indicate that preprocessing, wavelength selection, and model choice jointly affect classification performance for SWIR hyperspectral data [30]. In the binary task, SNV consistently yielded the best performance (e.g., SNV–MLP achieved 100% test accuracy; Table 3), likely because SNV reduces multiplicative scatter and improves spectrum-to-spectrum comparability under illumination and surface variability [7]. For the six-class staging task, SD produced the highest accuracy (SD–LDA: 92.56% test accuracy; Table 3), suggesting that second-derivative preprocessing enhances subtle local spectral differences and suppresses baseline drift, which is beneficial when separating adjacent contamination stages [10]. Feature wavelength selection further influenced multiclass recognition: UVE provided the most effective subset for six-class classification (UVE–LDA: 91.63% test accuracy; Table 4), implying that reliability-based elimination can preserve informative variables needed for fine-grained staging, whereas compact selections (e.g., SPA) may discard weak but relevant absorptions [36]. For binary detection under the controlled setting, several models achieved near-ceiling performance, while the six-class task remained more challenging due to gradual spectral evolution with contamination duration. Although the hybrid CLT model achieved high training accuracy, it did not consistently outperform simpler models on the test set (Table 3 and Table 4), suggesting sensitivity to model complexity and potential overfitting. The current conclusions are based on controlled inoculation/incubation with balanced categories, and broader validation under more diverse and field-like storage conditions is warranted.

## 4. Conclusions

This study focused on the accurate detection of *Gibberella zeae* contamination in maize kernels. A series of controlled inoculation and staged dynamic incubation experiments were conducted. Using SWIR hyperspectral imaging, the spectral response characteristics of maize kernels at different stages of fungal contamination were thoroughly analyzed, revealing the importance of specific wavelength bands in identifying *Gibberella zeae*. The effectiveness of various preprocessing techniques, including SNV, SD, and MSC, was systematically compared. Feature wavelength selection was further optimized using the UVE algorithm, and multiple classification models—such as LDA, MLP, and SVM—were established to accurately distinguish between healthy and contaminated samples at different stages. Among them, the binary classification task achieved 100% accuracy with the combination of SNV preprocessing and the MLP model, while the six-class classification task reached a high accuracy of 92.56% using SD preprocessing and the LDA model, demonstrating excellent classification performance. All data collected in this chapter were of high quality, with detailed traceability, further confirming the significant potential of hyperspectral imaging for early, rapid detection and classification of fungal contamination in maize kernels. These findings provide a strong theoretical foundation and data support for developing a reliable detection system for *Gibberella zeae* contamination in maize.

Although the modeling techniques evaluated in this study are established in the literature, our results provide comparative evidence on how different hyperspectral processing methods affect performance under a controlled early-contamination setting. Such evidence can support the design of future research and facilitate more robust method selection for practical grain quality monitoring.

## Figures and Tables

**Table 1:** System component information.

Component Name	Model	Manufacturer
Spectrometer	N25E	Specim, Spectral Imaging Ltd., Oulu, Finland
CCD Camera	XC-404	Xenics Infrared Solutions, Leuven, Belgium
Lens	OLES30	Xenics Infrared Solutions, Leuven, Belgium
Light Source	IRCP0078-1COMB	Isuzu Optics Corp., Taiwan, China
Stepper Motor Translation Stage	A49883-001	Isuzu Optics Corp., Taiwan, China

**Table 2:** Required equipment for experiments.

Instrument Name	Model	Manufacturer
High-pressure Steam Sterilizer	LDZX-50KBS	Shanghai Shen’an Medical Equipment Factory, Shanghai, China
Mold Incubator	MJ-250-1	Shanghai Yiheng Scientific Instrument Co., Ltd., Shanghai, China
Clean Bench	SW-CJ-2FD	Boxun Medical & Biological Instruments Co., Ltd., Shanghai, China
Electronic Balance	AL-104	Mettler-Toledo Instrument Co., Ltd., Greifensee, Switzerland
Biological Microscope	B302	Chongqing Aote Optical Instrument Co., Ltd., Chongqing, China
Electric Blast Drying Oven	DHG-9070A	Shanghai Yiheng Scientific Instrument Co., Ltd., Shanghai, China
Pipette	1-5000 μL	Eppendorf, Hamburg, Germany
Ultrapure Water System	LS-MK2	Pall Corporation, Port Washington, NY, USA

**Table 3:** Architecture summary of the proposed CLT model.

Module	Configuration
Input	Spectral vector reshaped to ((L,1))
CNN branch	Conv1D(64,k=3) → MaxPool(2) → Conv1D(128,k=3) → MaxPool(2) → Flatten
LSTM branch	LSTM(64)
Transformer branch	LayerNorm → MultiHeadAttention(heads=8, key_dim=64) → Dropout(0.1) → Flatten
Fusion + head	Concatenate → Dense(128, ReLU) → Softmax(6)

**Table 4:** Results of the maize kernel two-class and six-class task models under different preprocessing methods.

Preprocessing	Model	Six-Class Train Acc.	Six-Class Test Acc.	Two-Class Train Acc.	Two-Class Acc.
None	LDA	95.97%	92.21%	100.00%	100.00%
	PCA-LDA	55.18%	58.37%	81.50%	82.33%
	SVM	50.71%	53.49%	91.71%	93.26%
	CLT	97.75%	86.86%	100.00%	100.00%
	MLP	92.92%	87.21%	100.00%	100.00%
SG	LDA	95.13%	90.93%	100.00%	100.00%
	PCA-LDA	55.18%	58.49%	81.46%	82.21%
	SVM	49.87%	52.33%	91.71%	93.26%
	CLT	97.06%	88.49%	100.00%	100.00%
	MLP	95.58%	86.82%	100.00%	100.00%
SNV	LDA	95.60%	90.93%	100.00%	100.00%
	PCA-LDA	69.07%	70.23%	100.00%	99.88%
	SVM	62.45%	62.56%	100.00%	100.00%
	CLT	98.07%	89.77%	100.00%	100.00%
	MLP	98.17%	92.25%	100.00%	100.00%
FD	LDA	95.20%	91.51%	100.00%	100.00%
	PCA-LDA	68.05%	69.07%	100.00%	100.00%
	SVM	49.47%	51.98%	50.53%	48.02%
	CLT	99.96%	89.53%	100.00%	100.00%
	MLP	97.51%	87.21%	100.00%	100.00%
SD	LDA	95.46%	92.56%	100.00%	100.00%
	PCA-LDA	68.16%	69.07%	100.00%	100.00%
	SVM	49.47%	51.98%	50.53%	48.02%
	CLT	100.00%	89.65%	100.00%	100.00%
	MLP	99.83%	90.70%	100.00%	100.00%
MSC	LDA	95.38%	90.58%	100.00%	100.00%
	PCA-LDA	66.23%	69.07%	99.96%	99.88%
	SVM	49.55%	51.98%	100.00%	100.00%
	CLT	98.18%	89.19%	100.00%	100.00%
	MLP	98.54%	89.92%	100.00%	100.00%

Note: CLT denotes the hybrid CNN-LSTM-Transformer model.

**Table 5:** Results of maize kernel two-class and six-class task models under different feature wavelength selection methods.

Method	Model	Six-Class Train Acc.	Six-Class Test Acc.	Two-Class Train Acc.	Two-Class Acc.
CARS	LDA	85.82%	83.95%	100.00%	100.00%
	PCA-LDA	67.39%	68.60%	99.96%	100.00%
	SVM	49.47%	51.98%	100.00%	100.00%
	MLP	94.45%	81.40%	100.00%	100.00%
	CLT	98.98%	87.56%	50.53%	48.02%
SPA	LDA	73.10%	73.02%	100.00%	100.00%
	PCA-LDA	65.39%	66.98%	100.00%	99.88%
	SVM	49.47%	51.98%	100.00%	100.00%
	MLP	83.22%	73.26%	99.83%	99.61%
	CLT	93.31%	77.56%	100.00%	100.00%
UVE	LDA	95.38%	91.63%	100.00%	100.00%
	PCA-LDA	56.56%	60.00%	99.96%	99.88%
	SVM	49.84%	52.33%	100.00%	100.00%
	MLP	81.59%	77.91%	100.00%	100.00%
	CLT	95.17%	87.79%	100.00%	100.00%

## Data Availability

Data is contained within the article or Supplementary Materials. The original contributions presented in this study are included in the article/Supplementary Materials. Further inquiries can be directed to the corresponding authors.

## Supplementary Materials

The following supporting information can be downloaded at: https://www.mdpi.com/article/10.3390/s26061834/s1, Figure S1: CARS feature wavelength selection process and error variation; Figure S2: UVE feature wavelength selection process and error variation; Table S1: Feature wavelengths selected by UVE, SPA, and CARS methods for the classification of *Gibberella zeae* contamination in maize kernels.
