# Supplementary material for: Early Detection and Classification of Gibberella Zeae Contamination in Maize Kernels Using SWIR Hyperspectral Imaging and Machine Learning
Source: Sensors (Basel). 2026 Mar 14;26(6):1834. doi: 10.3390/s26061834 (PMC13030761; doi:10.3390/s26061834)
Supplement: Supplementary file 1 [file sensors-26-01834-s001.zip › sensors-4146122-supplementary.pdf]

Early detection and classification of *Gibberella zeae* contamination in maize kernels using SWIR hyperspectral imaging and machine learning

Kaili Liu [a,b,1], Shiling Li [a,1], Wenbo Shi [a], Zhen Guo [c], Xijun Shao [a], Yemin Guo [a,*], Jicheng Zhao [a,*], Xia Sun [a], Nortoji A. Khujamshukurov [d], Fangling Du [a,*]

[a] College of Agricultural Engineering and Food Science, Shandong University of Technology, No. 266 Xincun Xilu, Zibo, Shandong 255049, China

[b] Shandong Muyang New Energy Co. Ltd., Fulai Industrial Park, Rizhao, Shandong 276800, China

[c] State Key Laboratory of Macromolecular Drugs and Large-scale Preparation, Shandong Key Laboratory of Applied Technology for Protein and Peptide Drugs, School of Pharmaceutical Sciences and Food Engineering, Liaocheng University, Liaocheng 252000, China

[d] Department of Biotechnology, Tashkent Institute of Chemical Technology, Tashkent, 100011, Uzbekistan

[1] These authors contributed equally to this work.

[*] Corresponding authors: Professor Yemin Guo (E-mail: gym@sdut.edu.cn); Jicheng Zhao (E-mail: zhaojicheng@sdut.edu.cn); and Fangling Du (E-mail: fsmp@vip.126.com).

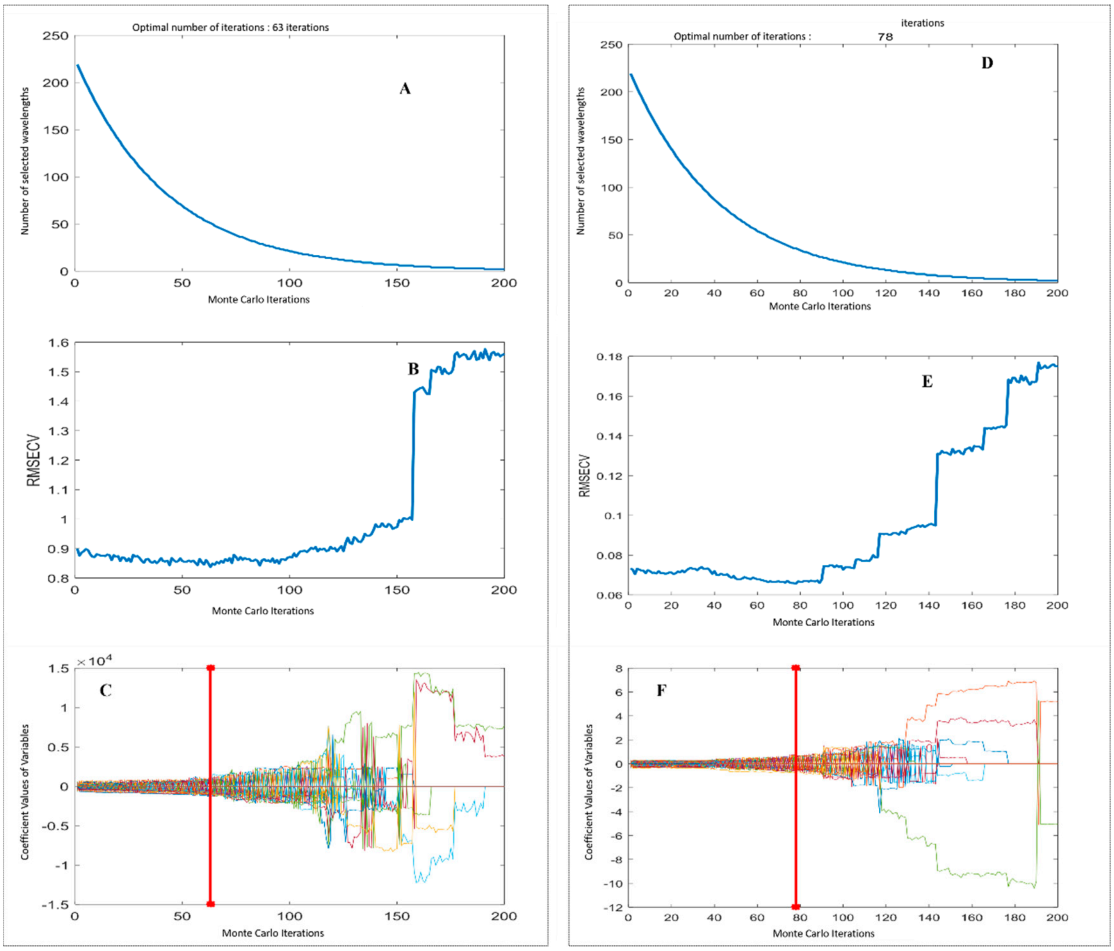

Fig. S1. CARS feature wavelength selection process and error variation, (A) Feature wavelength selection of the six-class task in CARS, (B) RMSE variation of the six-class task in CARS, (C) signal fluctuation of the six-class task in CARS, (D) feature wavelength selection of the two-class task in CARS, (E) RMSE variation of the two-class task in CARS, (F) signal fluctuation of the two-class task in CARS

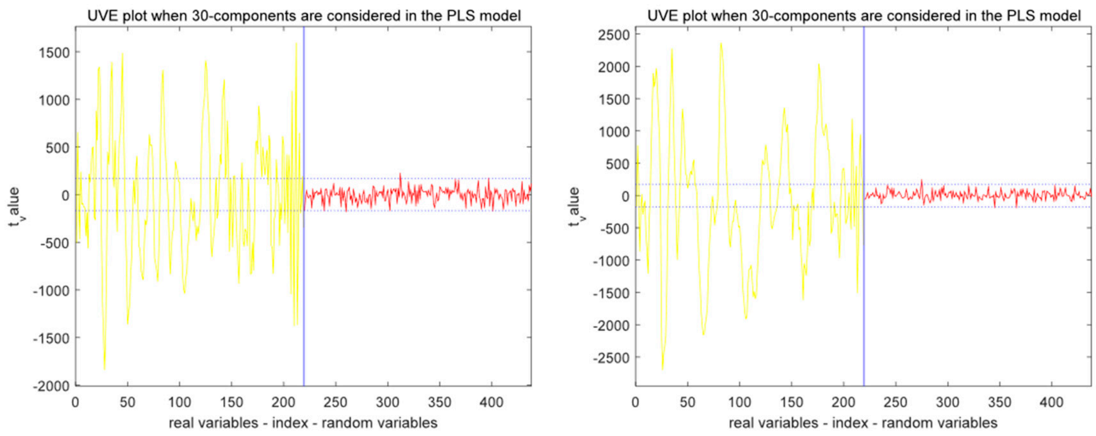

Fig. S2. UVE feature wavelength selection process and error variation, (A) Feature wavelength selection of UVE for six-class task, (B)feature wavelength selection of UVE for two-class task

Table S1.

Feature wavelengths selected by UVE, SPA, and CARS methods for the classification of *Gibberella zeae* contamination in maize kernels.

Method	Task	Number of Selected Wavelengths	Selected Wavelengths (nm)
SPA	Two-class	17	901, 926, 957, 1007, 1064, 1114, 1196, 1409, 1446, 1563, 1697, 1780, 1803, 1838, 1873, 1930, 2201
CARS	Two-class	30	939, 1001, 1039, 1058, 1064, 1070, 1114, 1290, 1296, 1465, 1551, 1557, 1774, 1780, 1862, 1902, 1925, 1930, 1936, 1970, 1975, 1998, 2003, 2014, 2080, 2138, 2144, 2154, 2165, 2170
UVE	Two-class	140	901, 907, 920, 926, 957, 964, 970, 976, 989, 995, 1001, 1007, 1014, 1020, 1026, 1033, 1039, 1045, 1051, 1058, 1064, 1070, 1077, 1083, 1089, 1095, 1102, 1108, 1114, 1121, 1133, 1146, 1152, 1158, 1165, 1171, 1177, 1183, 1190, 1196, 1202, 1209, 1215, 1221, 1227, 1234, 1259, 1265, 1278, 1284, 1290, 1296, 1303, 1309, 1316, 1321, 1328, 1334, 1340, 1346, 1353, 1359, 1371, 1378, 1384, 1390, 1396, 1409, 1415, 1421, 1428, 1434, 1440, 1459, 1465, 1471, 1477, 1483, 1496, 1502, 1508, 1526, 1533, 1539, 1545, 1551, 1557, 1563, 1569, 1575, 1582, 1594, 1600, 1612, 1618, 1624, 1630, 1636, 1648, 1654, 1660, 1667, 1673, 1679, 1685, 1691, 1697, 1703, 1709, 1715, 1720, 1726, 1732, 1738, 1744, 1750, 1756, 1762, 1768, 1774, 1780, 1792, 1797, 1803, 1809, 1815, 1827, 1833, 1838, 1850, 1856, 1862, 1867, 1879, 1885, 1890, 1896, 1902, 1908, 1925, 1930, 1936, 1942, 1947, 1953, 1959, 1964, 1970, 1975, 1981, 1987, 1992, 1998, 2003, 2009,

			2014, 2025, 2031, 2036, 2042, 2047, 2053, 2058, 2064, 2080, 2091, 2096, 2101, 2107, 2117, 2123, 2128, 2138, 2144, 2154, 2159, 2165, 2170, 2175, 2180, 2185, 2195, 2201
SPA	Six-class	13	1070, 1551, 1691, 1774, 1797, 1896, 1992, 2036, 2058, 2080, 2101, 2170, 2185
CARS	Six-class	40	1045, 1070, 1077, 1083, 1108, 1165, 1171, 1221, 1265, 1321, 1328, 1334, 1340, 1346, 1421, 1427, 1434, 1612, 1618, 1624, 1630, 1673, 1679, 1756, 1762, 1768, 1774, 1780, 1786, 1815, 1821, 1879, 1981, 1987, 2042, 2096, 2128, 2175, 2180, 2185
UVE	Six-class	151	907, 920, 926, 939, 957, 964, 970, 976, 989, 995, 1001, 1007, 1014, 1020, 1026, 1033, 1039, 1051, 1058, 1064, 1070, 1077, 1083, 1089, 1095, 1102, 1108, 1114, 1121, 1127, 1133, 1139, 1146, 1152, 1158, 1165, 1171, 1177, 1183, 1190, 1196, 1202, 1215, 1221, 1227, 1234, 1240, 1246, 1271, 1278, 1284, 1290, 1296, 1303, 1309, 1315, 1321, 1328, 1334, 1340, 1346, 1365, 1371, 1378, 1384, 1396, 1403, 1409, 1415, 1421, 1427, 1434, 1440, 1446, 1459, 1465, 1471, 1477, 1483, 1489, 1502, 1508, 1514, 1520, 1526, 1533, 1539, 1545, 1551, 1557, 1563, 1569, 1575, 1582, 1588, 1594, 1600, 1606, 1612, 1618, 1624, 1630, 1636, 1648, 1654, 1660, 1667, 1673, 1679, 1685, 1691, 1697, 1703, 1709, 1715, 1720, 1726, 1732, 1750, 1756, 1762, 1768, 1774, 1780, 1786, 1792, 1797, 1803, 1827, 1838, 1844, 1862, 1867, 1873, 1879, 1885, 1890, 1896, 1902, 1908, 1919, 1925, 1942, 1947, 1953, 1959, 1964, 1970, 1975, 1981, 1987, 1992, 1998, 2003, 2009, 2014, 2020, 2025, 2036, 2042, 2047, 2058, 2064, 2069, 2074, 2080, 2091, 2096, 2101, 2107, 2112, 2117, 2123, 2133, 2138, 2144, 2149, 2154, 2165, 2170, 2175, 2180, 2185, 2190, 2195, 2201
